# Research on the Oscillation in Centerless Grinding Technology When Machining Bearing Steel

**DOI:** 10.3390/ma15144968

**Published:** 2022-07-17

**Authors:** Martin Gavlas, Michal Kaco, Vladimír Dekýš, Miroslav Špiriak, Silvia Slabejová, Andrej Czán, Jozef Holubjak, Milena Kušnerová, Marta Harničárová, Jan Valíček

**Affiliations:** 1 Department of Applied Mechanics, Faculty of Mechanical Engineering, University of Žilina, Univerzitná 8215/1, 010 26 Žilina, Slovakia; martin.gavlas@fstroj.uniza.sk (M.G.); michal.kaco@fstroj.uniza.sk (M.K.); vladimir.dekys@fstroj.uniza.sk (V.D.); 2 Department of Machining and Manufacturing Technologies, Faculty of Mechanical Engineering, University of Žilina, Univerzitná 8215/1, 010 26 Žilina, Slovakia; miroslav.spiriak@fstroj.uniza.sk (M.Š.); silvia.slabejova@fstroj.uniza.sk (S.S.); andrej.czan@fstroj.uniza.sk (A.C.); jozef.holubjak@fstroj.uniza.sk (J.H.); valicek.jan@mail.vstecb.cz (J.V.); 3 Department of Mechanical Engineering, Faculty of Technology, Institute of Technology and Business in České Budějovice, Okružní 10, 370 01 České Budějovice, Czech Republic; kusnerova.milena@mail.vstecb.cz

**Keywords:** centerless grinding, vibrodiagnostics, analysis of the run-up and run-down, boxplot, spectral analysis, STFT, geometric deviation

## Abstract

In today’s engineering industry, technical diagnostics presents many advantages for improving the management of machining centers and automated production lines. As the fourth industrial revolution is currently being implemented, which includes machine diagnostics, the idea of adding information from the field of vibrodiagnostics was born. The vibration of the workpiece or machine tool negatively affects the geometric parameters of the machined surfaces of the workpiece. Through vibrodiagnostics, the influence of cutting parameters on the oscillation of a bearing steel workpiece during centerless grinding is investigated. The presented publication deals with the vibration of the mechanical parts of a centerless grinding machine. The oscillations are recorded by acceleration sensors, which are also placed on the support ruler in which the workpieces are guided, and the recorded data are input parameters for statistical processing of acceleration values in the form of statistical characteristics (minimum, lower quartile, median, upper quartile, maximum). In this paper, this procedure was applied for the selection of the optimum cutting parameters (for the speed of the support wheel), where the machining parameters at which the minimum oscillation values occur were selected based on the above-mentioned statistical characteristics. This optimization procedure revealed increased vibration values which reached the highest amplitude on the ruler, namely accelerations of 11 m/s^2^, the origin of which was subsequently detected by STFT because the occurrence of resonance events or the excitation of natural frequencies of the machine were suspected. The STFT analysis identified a resonant region at machine start-up determined by the spindle speed which excites the resonance on the machine. The speed range between 1950 and 2150 rpm, which corresponds to the built-up resonance, was provided to the technologists to ensure that the machine was not operated around this resonance region at 400 and 760 Hz until the undesired phenomenon was eliminated. The results of the individual measurements provided information on the ideal setting of the cutting parameters and the current state of the machine.

## 1. Introduction

Mechanical production, machining, and technical diagnostics are accompanied by developments and various innovations, the main task of which is to surpass previous goals. This production must be in a constant cycle of innovation, as it is directly affected by developments and innovations in various technical materials. The current trend in engineering is to apply the knowledge gained from previous developments to current issues to the largest possible extent and thus gain a competitive advantage over other market players.

Technical diagnostics is currently one of the major disciplines. It is composed of sets, procedures, and activities that can be used to prevent unexpected faults or downtime. It can be applied in all areas of technology and industry to ensure product quality, make processes more efficient, and above all, improve safety and reliability [1]. Automated machining lines cover many process operations. These are dependent on the correct specifications of the tool, workpiece, the correct choice of machine tool, as well as machining method and cutting parameters [2]. The above-mentioned factors can be considered as variables, and they further influence other factors that are involved in the correct tool wear, the magnitude of cutting forces, the magnitude of vibrations generated, the temperature generated during the cutting process, and also the surface quality of the workpiece (e.g., dimensional accuracy, the surface quality of the workpiece due to machining, etc.) [3].

Technical diagnostics methods are differentiated according to the different monitored physical variables, which allow for determining the optimal condition of the monitored object. Currently, the operator performing machine maintenance can choose the correct method of technical diagnostics to determine the current state of the equipment. The selection of the method is based on several criteria. The first criterion is the machine design system. The variety of technological processes influences the approach to the diagnosis of the structural parts from which information on the components in question is obtained. The second criterion is the type of output required. By elaborating the criteria, the appropriate type of method can be determined to obtain the necessary information for evaluating the condition of the equipment [4].

Various sensors are used to record the information, and their role is to record variable factors (temperature, vibrations, forces, etc.). That is to say, they monitor any abnormalities that occur in the system, and based on the information obtained, are able to prevent unexpected anomalies, which help to prolong the life of the system, machine, and tool [5].

Oscillations in the machining process are among the undesirable phenomena because they have an adverse impact on the mechanical parts of the machine, reduce its lifetime, also reduce the quality of the surface of the workpiece, and damage the machined tool, as the geometry of the workpiece is changed. These oscillations can be recorded using accelerometric sensors, which are able to determine the dynamic acceleration at the measuring point, which can transmit the oscillations of the rotating tool, workpiece, etc. The advantage of accelerometers is their wide measuring range, which makes it possible to identify the machining process. The disadvantages of accelerometers are lower sensitivity and loss of data in the high-frequency range. In machining processes, cutting-edge degradation and reduction of workpiece surface roughness occur [6].

A large part of emerging faults can be detected in advance, as they are manifested by the damaged objects. Therefore, by implementing predictive maintenance to predict the condition of the machine in real time, it is possible to achieve a reduction in downtime on a given machine [7]. Vibrodiagnostics is often used in practice and is, therefore, one of the most common methods for monitoring the technical condition of rotating machinery. By performing vibrodiagnostics, machine faults can be identified. By eliminating the identified faults, the aforementioned aspects are again ensured [8,9].

The methods of technical diagnostics have developed in connection with the automotive industry, as many technological devices are used in the production of individual components. In the automotive industry, it is also necessary to ensure the high-precision, high-volume production of individual parts. Various finishing machining methods achieve high precision [10]. These finishing methods no longer reshape the overall geometry but only refine the resulting properties. Therefore, the grinding process must ensure the correct profile of the geometric parameters [11].

As grinding technology is often at the end of the manufacturing process [12], it is necessary to capture oscillations and minimize their occurrence due to the fact that they may end up affecting the workpiece and causing rejects. By reducing the oscillations in grinding, a reduction in financial losses will be achieved because these rejects have already passed through certain machining operations [13].

**Figure 1:**
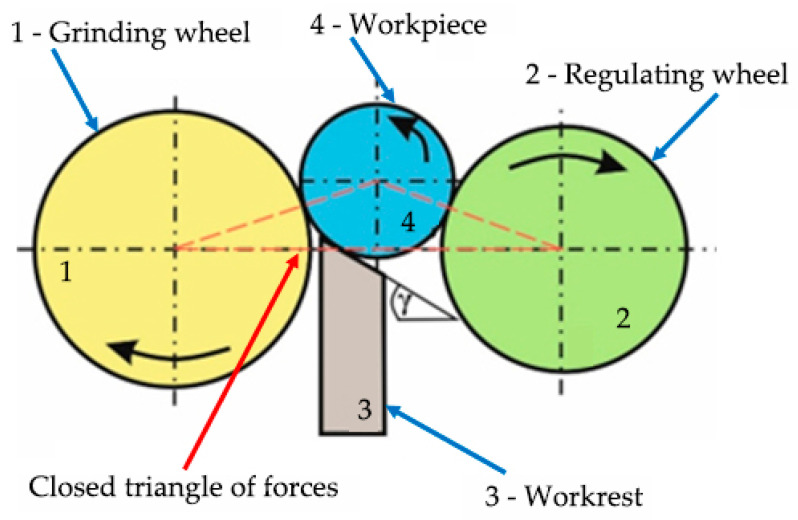
Centerless grinding technology—horizontal type.

Centerless grinding uses the technology of the workpiece support system and the workpiece rotation drive mechanism (Figure 1).

The principle of centerless grinding on a machine tool, i.e., on a centerless grinder, is that the rotating surfaces of the workpiece are ground when inserted between two rotating wheels. One wheel has a grinding function, i.e., it performs the main grinding process of the workpiece (grinding wheel). The second wheel (regulating wheel) has a carrier function, and its influence regulates the rotation of the workpiece. The rotation of the workpiece is also influenced by the positioning of the workpiece between the axes of the wheels, and the workrest helps to maintain the correct positioning [14]. Based on the geometric position of the grinding wheel, the regulating wheel, and the workpiece itself, we find a closed triangle of forces (marked with a red dashed line in Figure 1); these forces help us to guide the workpiece flawlessly and safely throughout the grinding cycle. The machine tool, the grinding tool, and the workpiece form a machining system with complex dynamic characteristics, in which the oscillation of the individual elements is a concomitant of the cutting process. Centerless grinding technology, as we know it today, was introduced in 1917 on the basis of a Heim patent. The patent describes the initial concept and design of an early machine [15]. Among the first designers in Europe was the Swedish company Lidköpings Mekaniska Verkstads (LMV) [16]. The subsequent development of centerless grinding was accelerated by the high demand for various components in the developing automotive industry.

A review study by Hashimoto demonstrates advances in centerless grinding. It also highlights the complexity of the centerless grinding technology used and the influence of various technological factors on the resulting workpiece quality. The aim of his study is to provide an overview of centerless grinding technology, the design of critical machine elements, and the definition of guide lines for the design of future machines [17].

Centerless grinding technology is very sensitive to the cutting parameter setting conditions. If the cutting conditions are not set correctly, various problems such as increased oscillation, unsatisfactory workpiece parameters, workpiece drift problems, etc., can occur. Several studies show that the rigidity of the system greatly influences the geometrical parameters of the workpiece. The instability of the system induces oscillations that directly affect the workpiece [18,19].

In order to meet the demand, centerless grinding technology has also met the need to increase the productivity of grinding machines. This increase in productivity can be achieved by changing the cutting conditions. A study dealing with productivity enhancement points out the disadvantages associated with changing the cutting conditions by changing the position of the workpiece. This change can achieve a reduction in the time of the grinding operation. Nevertheless, the main disadvantage of changing the workpiece position is that it changes the rotational speed of the workpiece, affecting the machining operation’s stability. Moreover, the instability of the system affects the resulting geometry of the workpiece [20,21]. The effects of workpiece displacement relative to the cutting tool axis are also discussed by other researchers in their studies [22,23].

Research dealing with the grinding regulation and regulation wheel axes in combination with a suitable carrier profile can also be included in these studies. The angle and position of the workpiece cannot be regulated independently because their relationship is defined in the blade design. As a result of the research, the optimum choice of machining parameters can be made, which also makes it possible to reduce machining times [24].

The input shape of the workpiece can also cause the oscillation of the system. It may contain some polygons that can be well simulated and their critical number recalculated. Simulations further present that the unstable state of the system is the result of a moving workpiece with a polygonal shape and a vertical displacement of the workpiece between the grinding and regulating wheel [25].

**Figure 2:**
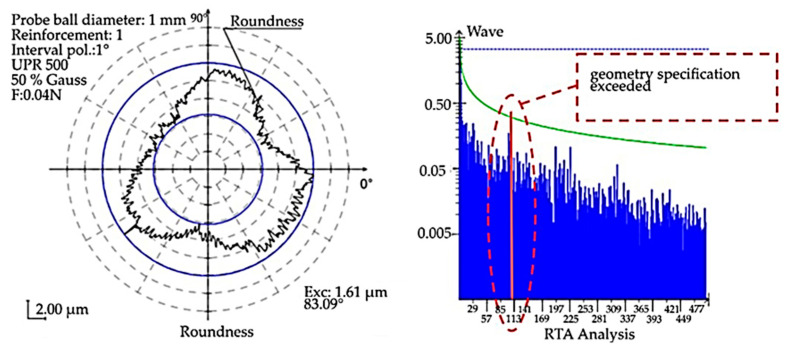
RTA analysis showing over-specification.

A review of published work on centerless grinding shows that oscillations in the centerless grinding process can be directly influenced by changes in cutting parameters. Therefore, the motivation of this paper is to identify possible changes in oscillation under different cutting conditions on the Nomoco VSR-5-280 technological system in the experimental part. The reason for the implementation of the machine oscillation diagnostics is that the back-check of the fabricated parts showed a high formation of non-defects (Figure 2). The RTA analysis shows an exceedance of the established specification (in terms of surface geometry and functionality). Suppose the RTA orbit analysis shows such an over-specification of geometrical parameters with the parameters prescribed in the technical drawing of the workpiece. In that case, it is assumed that there may be other non-compliance with the required geometrical specifications in the given workpiece as well. Such a workpiece is considered unsuitable and must be discarded. The changes in the machine set-up during grinding made by the technologist and the machine operator based on their experience were not sufficient to achieve the required geometric parameters. Therefore, other possibilities influencing the development of unsatisfactory workpiece geometric parameters were investigated. Based on the above-mentioned search, it is concluded that unrealized experimental procedures have been defined, which consist in changing the cutting parameters, i.e., changing the speed of the support wheel in the range of +25% to −25% stepped in 5% increments. The machine uses centerless grinding technology, which is susceptible to workpiece oscillation during grinding. The oscillation generated during grinding is considered to be the dominant factor affecting the fulfilment of the required geometric parameters. Since geometric parameters can be achieved at different cutting conditions, several modes with different cutting conditions were selected, and it was necessary to assess the degree of oscillation. Based on the minimum oscillation values, the optimum grinding mode was supposed to be selected.

## 2. Materials a Methods

### 2.1. Presentation of the Equipment Used in the Experimental Measurement and the Workpiece Material

**Figure 3:**
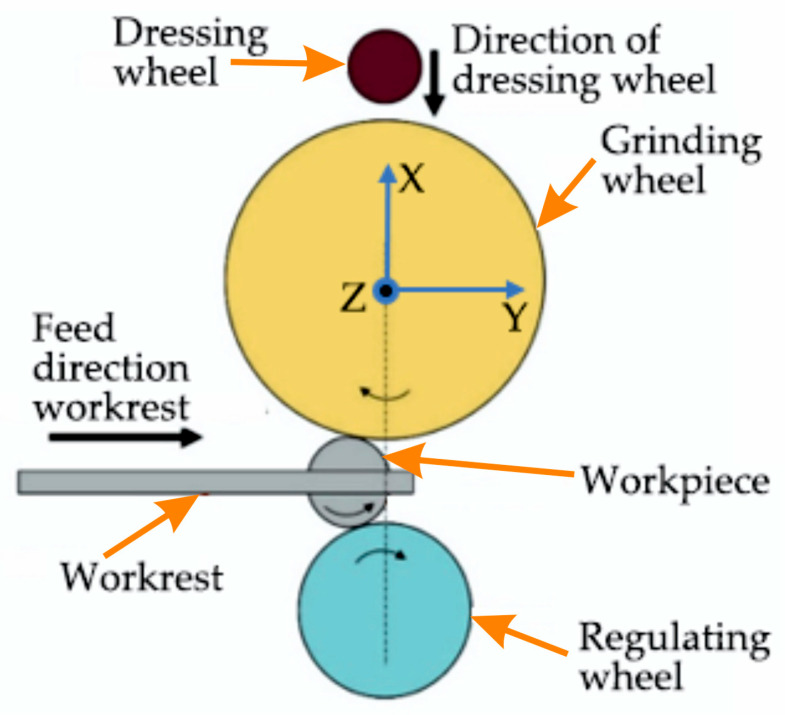
Technological diagram of the Nomoco VSR-5-280.

The experimental measurement was carried out on a Nomoco VSR-5-280. This machine belongs to the vertical type of centerless grinding machine (Figure 3), where the blue arrows show the coordinate axis of the moving parts. The “*X*” axis is oriented vertically; the wheels (grinding, workrest, and dressing wheels) also move in this direction; that is why the technological scheme is called “vertical”. The “*Y*” axis is oriented horizontally, and the workrest moves in this direction together with the workpieces. The aforementioned coordinate axis in Figure 3 coincides with the axis of the sensors on the Nomoco device.

The grinding wheel which performs the main working function is defined by the designation 3SK3 120 J0T7 V09G, where:

- 3SK3 is sintered aluminum oxide, vitreous
- 120 defines the grain size of the wheel, i.e., fine grain (specified by DIN ISO 8486-1).
- T7 describes the hardness and concentration of the grinding wheel, where “T” is the hardness—very hard, and No. 7 defines the concentration of the abrasive in the grinding body.
- V09 defines the bonding agent, i.e., the standard high-performance bonding agent for microcrystalline corundum.

**Figure 4:**
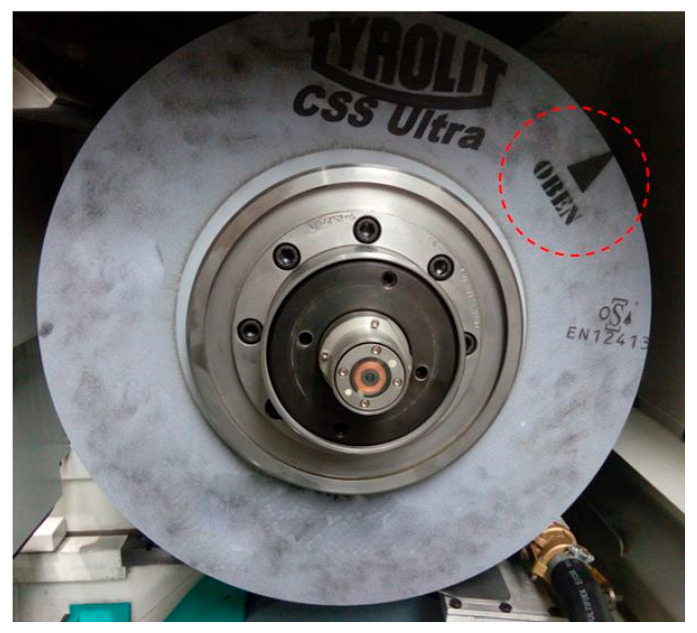
Illustrative picture of the grinding wheel after balancing with the “OBEN” point marked, indicating the correct orientation.

Before the start of the actual experiments, a check balancing of the grinding wheel was carried out to achieve high dimensional accuracy and surface quality of the workpiece and reduce the wear of the grinding spindle bearings. Suppose the center of gravity of the wheel coincides precisely with its geometric center. In that case, the axis of rotation and unbalanced forces are eliminated, and the wheel rotates evenly even at high peripheral speeds. The balancing of the grinding wheel is carried out by a machine that uses electromagnetic force for balancing. Based on the results obtained from the balancing machine, a location is marked on the grinding wheel (Figure 4), which indicates the orientation of the wheel with respect to the vertical axis.

Brüel and Kjær piezoelectric accelerometers type 4507-B-004 with standard technical parameters were used to record the oscillations (Table 1). The sensors are uniaxial, i.e., they record information in one direction. The individual accelerometers were connected to a cDAQ 9234 A/D converter (National Instruments—NI).

A Pocket Laser Tachometer 100 (Monarch) connected to an NI cDAQ 9234 unit was used to record the speed.

All A/D input modules were housed in a cDAQ 9178 (NI) chassis. Information acquisition was controlled by the LabView-SignalExpress (NI) program, and data processing was performed in DIAdem (NI).

**Figure 5:**
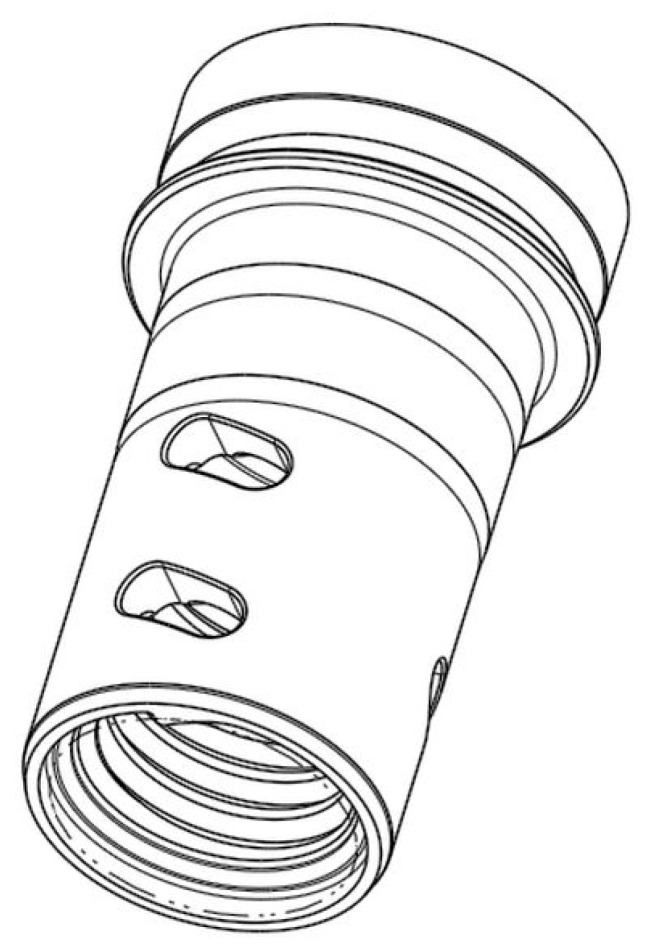
A workpiece being machined.

The machined element (Figure 5) was made of bearing steel and was in the post-heat-treated condition. The chemical composition of the material can be found in Table 2 and is consistent with the material data sheet. The material data sheet refers to EN ISO 683-17. The standard also describes the tensile strength, which after heat treatment (quenching and annealing) reaches values between 570 and 780 MPa. The hardness of the workpiece is defined by the chemical composition of the bearing steel, which reaches the specified hardness of ~62HRC after heat treatment. That is, the measurement is made by the Rockwell hardness measurement method. The hardness measurement was carried out after each heat treatment on ten samples. From the measured values, the average value declared above is taken with an accuracy of ±5%.

Bearing steels are classified as structural steels according to their use, even though their chemical composition and thus their properties correspond more closely to those of tool steels. In addition to technical parameters, bearing steels must also meet high technological requirements. These include, in particular, good and uniform machinability and adequate machinability, while simultaneously dimensional stability of the finished bearings. The machinability of the bearing steel is also affected by inclusions that may be present in the workpiece, and during finishing (grinding, superfinishing, etc.), these inclusions are rolled out, and surface defects occur on the functional surfaces. Therefore, in bearing steels, high emphasis is placed on the correct chemical composition and the purity of the material structure. The workpiece is manufactured from bearing steel because it contains functional surface areas over which the bodies roll in a fully assembled state.

### 2.2. Description of the Grinding Process, Changes in Cutting Parameters and Measurement Procedure

The grinding process consisted of grinding the outer rotating area, which contains a surface with the required geometric parameters. The grinding process consisted of several successive operations:

Operation 1—preparatory phase:

- Loading three workpieces simultaneously into the ruler (the carrier—workrest, which provides guidance and positioning of the workpieces during grinding);
- Moving the workpiece ruler to the machining site between the grinding wheel and the regulating wheel; these wheels carry out the grinding process.

Operation 2—grinding process phase.

Operation 3—completion phase.

After the finishing phase, the unloading process is carried out, i.e., the ruler is moved to the workpiece unloading location. After unloading the workpieces (machined pieces), the ruler is moved to the position for carrying out the preparation phase.

The stages of the grinding process were characterized by common and different cutting conditions in the selected regimes. Table 3 lists some basic cutting conditions selected from a group of common conditions that characterize the grinding process.

The different cutting conditions are based on varying the initial speed of the regulating wheel from −25% to +25% in increments of 5%. The selected interval for changing the speed of the support wheel was chosen based on the long experience with similar machining materials on the same machines in the company. Speeds outside this interval can cause faster wear of the grinding wheel; wear on the mechanical parts of the machine increases. Moreover, such interaction between the rotating grinding wheel and the workpiece may lead to thermomechanical stresses in the contact zone, which may cause undesirable effects on the material properties of the workpiece [26]. Table 4 shows the different cutting conditions, which also specify the grinding modes for the measurements. The variation of the cutting conditions characterizes one mode, i.e., 11 modes were implemented, indicated by the numbers: −25, −20, −15, −10, −5, 0, +5, +10, +15, +20, +25. The original speed of the dressing wheel is denoted by the number 0.

**Figure 6:**
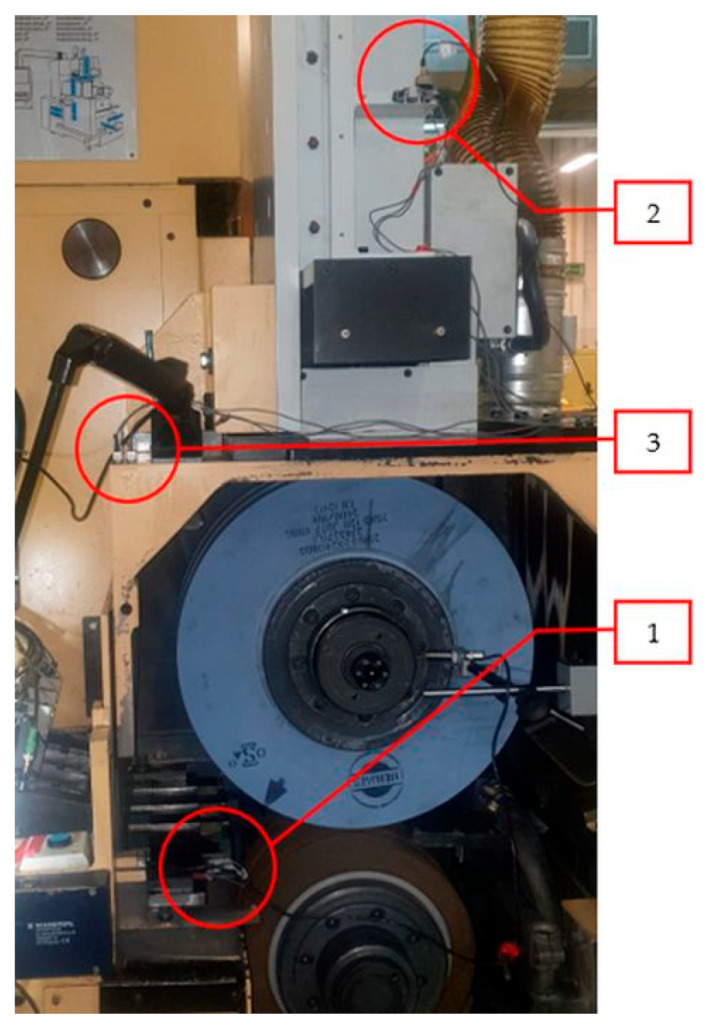
Location of sensors on the Nomoco VSR-5-280 machine with three locations, position 1—ruler, position 2—frame of the dressing wheel and position 3—machine frame.

Sensors were placed at three locations (Figure 6):

1. Ruler,
2. The frame of the dressing wheel,
3. Machine frame.

At each site, the sensors were oriented in three x, y, z directions.

For both parts of the measurements, all positioned sensors were used in positions 1, 2, 3 (Figure 6). In each position, there were three uniaxial accelerometers for the directions “x, y, z”—i.e., nine sensors in total. The data acquisition from the accelerometers and the tachometer was simultaneous at a sampling rate of 10 kHz/channel. The measurement process was initiated before the measurement started, and the subsequently evaluated stationary mode was selected using a software trigger. Within one mode, two measurements were performed on two groups of workpieces (these measurements are distinguished in the displays as Grinding 1 and Grinding 2).

The measurement was divided into two parts.

**Figure 7:**
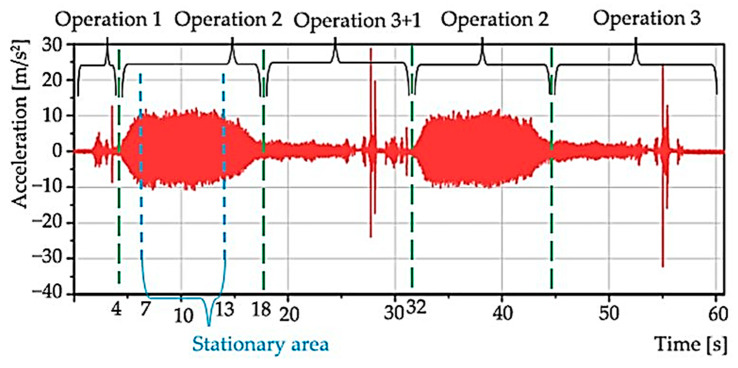
Acceleration values from one whole mode measurement.

**The first measurement** consisted of measurements in each mode (Table 4), where two grinding processes were performed for each mode. The acceleration values of one of the waveforms allow a good recognition of the non-stationary nature of the recorded random process realization (Figure 7). The latter can be divided into operation 1 (up to approximately 4 s), operation 2 (from approximately 4 to 18 s), operation 3 + 1 (from 18 to 32 s), and so on.

**Figure 8:**
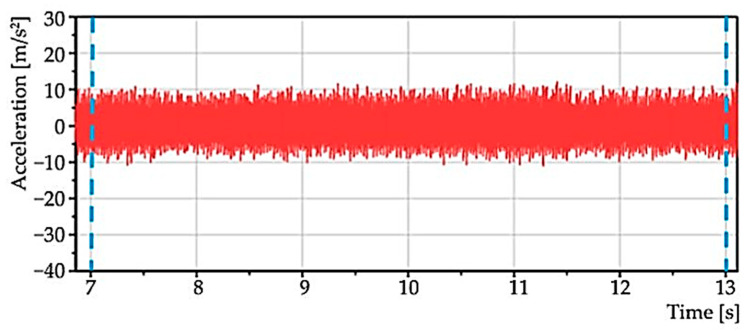
Zoom of the stationary area with the previous figure.

From operation 2, a stationary part was selected—zoom (Figure 8) in a time interval of 6 s; in Figure 6, the interval is from 7 to 13 s. It is from this area that the statistical characteristics were determined.

Statistical characteristics:

- minimum (*x_min_*),
- 0.25 quantile—lower quartile (*x*_0.25_),
- 0.50 quantile—median (*x*_0.50_),
- 0.75 quantile upper quartile (*x*_0.75_),
- maximum (*x_max_*).

The statistics were calculated based on the following equations:

(1) $$x_{min}=x_{0.25}-1.5*IQR$$

(2) $$x_{max}=x_{0.75}-1.5*IQR$$

where *IQR* is the interquartile range (3):

(3) $$IQR=x_{0.75}-x_{0.25}$$

Based on these values, the different grinding modes were compared. The aim of this measurement was to select a suitable mode in which minimum oscillation occurs.

**The second measurement** was also made because this machine behaved anomalously when compared to the same machines in the production plant. Therefore, based on experience, it was decided to also perform further analysis, under run-up and run-down machine conditions. In this mode, there was no grinding but only run-up and run-down of the grinding wheel. This measurement aimed to exclude or confirm the occurrence of a resonance region that could be excited by the residual unbalance of the rotating grinding wheel.

## 3. Analysis of Measured Data

### 3.1. Processing for Individual Modes

**Figure 9:**
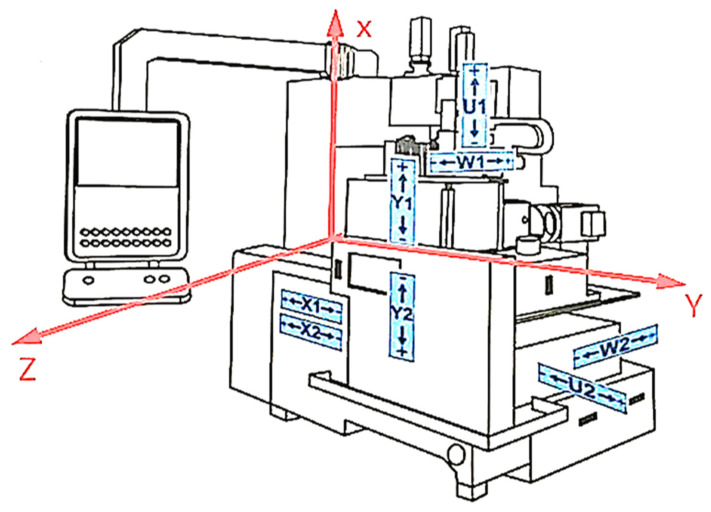
Schematic drawing of the sensor axis directions to the Nomoco device.

**Figure 10:**
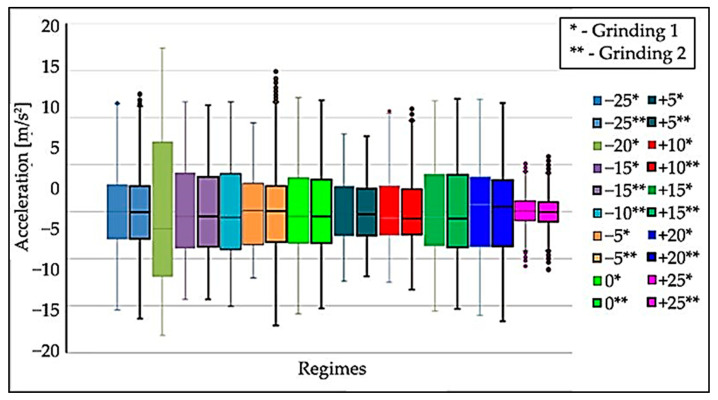
Boxplots of the acceleration of the oscillation in the tested modes on the ruler in the “*x*-axis” direction, grinding wheel speed—1907.2 [rpm], basic speed (regime 0) for regulating wheel—20.2 [rpm], workpiece speed—254 [rpm], ruler distance—13 [mm].

**Figure 11:**
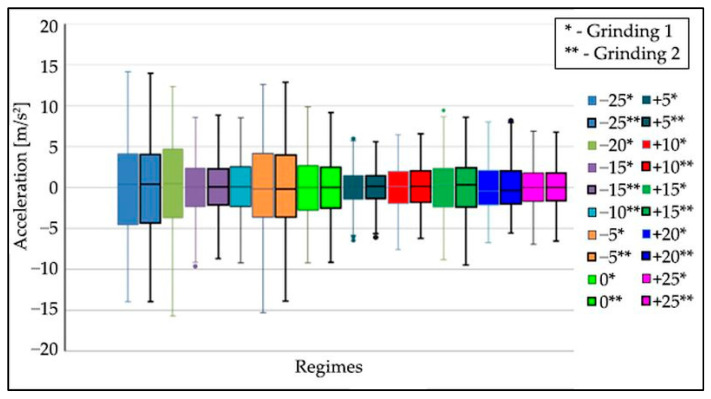
Boxplots of the acceleration of the oscillation in the tested modes on the ruler in the “*y*-axis” direction, grinding wheel speed—1907.2 [rpm], basic speed (regime 0) for regulating wheel—20.2 [rpm], workpiece speed—254 [rpm], ruler distance—13 [mm].

**Figure 12:**
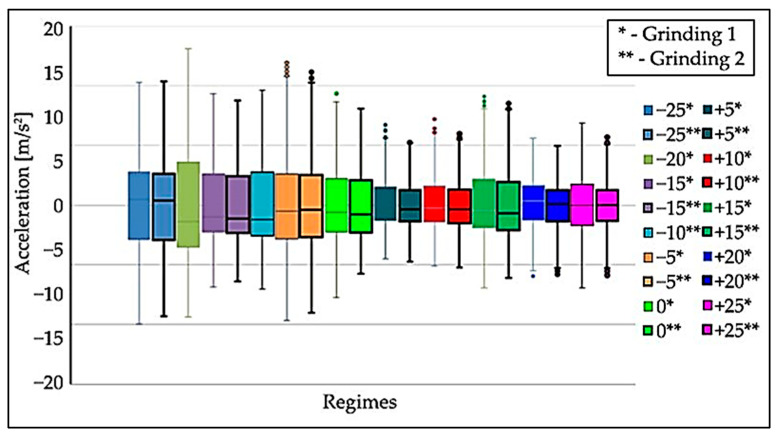
Boxplots of the acceleration of the oscillation in the tested modes on the ruler in the “*z*-axis” direction, grinding wheel speed—1907.2 [rpm], basic speed (regime 0) for regulating wheel—20.2 [rpm], workpiece speed—254 [rpm], ruler distance—13 [mm].

From the first measurement, the statistical characteristics (*x_min_*, *x_max_*, *x*_0.25_, *x*_0.5_, *x*_0.75_) were plotted in graphs using boxplots. Based on the generated boxplots, a quick comparison of the measurements of each mode was performed. Schematic drawing of the sensor axis directions to the Nomoco device is shown in Figure 9. Figure 10, Figure 11 and Figure 12 show the boxplots for the *x*, *y*, *z* axes. For easier determination of the correct results, tables were created.

Table 5 shows the colour coding of the different grinding modes used in the following figures. 

Each mode contains two grinding operations with the same input parameters. The different modes (Figure 10, Figure 11 and Figure 12) are distinguished by different colors; the operations in one mode have the same color but differ in the border.

When processing the data after the measurements, it was found that the −20% and −10% mode files did not contain the correct data due to measurement error, so there is only one boxplot each for these measurements in the boxplots in Figure 10, Figure 11 and Figure 12.

Data analysis showed that the highest values of oscillation amplitudes are at the feed ruler in the x-direction. These values exceeded the acceleration of 11 m/s^2^. Therefore, the data from the feed ruler was processed to determine the non-compliant mode accurately.

For a more detailed determination of the compliant mode, the percentage value of the quartile range *x*_0.75_–*x*_0.25_ was calculated for all modes, with the range corresponding to mode 0 considered as the reference range. These data can be found in Table 6.

**Figure 13:**
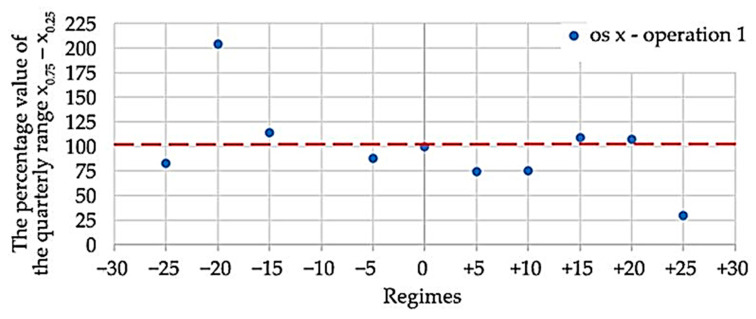
The percentage value of the quarterly range *x*_0.75_–*x*_0.25_.

For better interpretation, the data are converted into a graphical representation (Figure 13), which contains the values (blue points) belonging to operation 1 for the *x*-axis. Since the difference between the operations is minimal, for simplicity of the plot, only one operation was selected, namely in the axis where the oscillation values were the highest. The dashed red line shows the reference mode, and points outside this value show the deviation from the reference mode. Based on this data, it is evident that the most optimal cutting conditions are in the +25 mode, where a drop in oscillation values of up to 50% occurred. At −20 mode, the oscillation values increased by more than 50%.

The percentage value of the quarterly range *x*_0.75_–*x*_0.25_ depending on the selected modes (Figure 13).

The following conclusions can be drawn from the processing of the measurement data:

1. The differences between the oscillation level at the first and second operations in the modes with the same parameters are negligible.
2. The effect of the changed speed of the dressing wheel causes the ruler to oscillate with the workpieces. The highest oscillation values were recorded in the direction of about x.
3. The lowest oscillation values were registered in the −25 mode, the most optimal mode.

### 3.2. Analysis of the Run-Up and Run-Down

By the first measurement, it was found that the highest values of the oscillation amplitudes are on the feed ruler in the x-direction. The values of the oscillation amplitudes exceeded the acceleration of 11 m/s^2^. Therefore, the following part of the analysis is focused on the channels corresponding to this direction.

**Figure 14:**
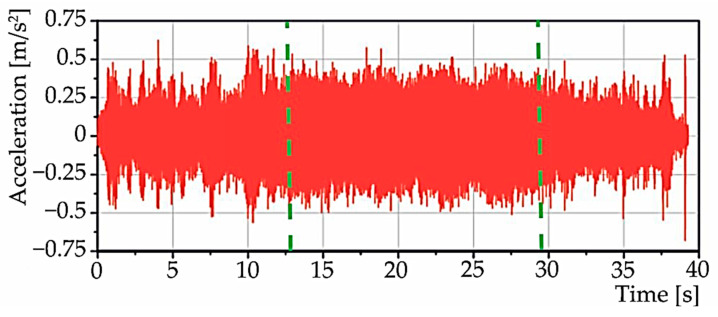
Time waveform of the signals when the machine spindle starts to accelerate (simultaneous data acquisition).

**Figure 15:**
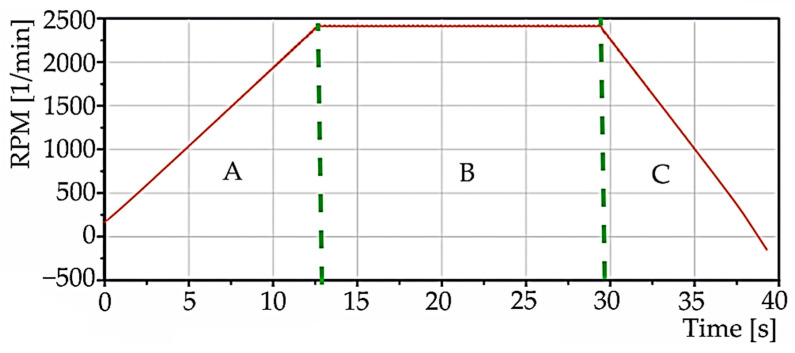
Time waveform of signals during machine spindle start-up for speed (simultaneous data acquisition).

Figure 14 and Figure 15 show the data recorded at simultaneous data acquisition, acceleration on the ruler in the x-direction, and speed. The figures are divided into three regions: A, B, C. The first region, “A”, belongs to the run-up of the selected speed, i.e., 2500 rpm. The second region, “B”, belongs to staying at the selected speed. The third region, “C”, records the decrease of the speed to zero.

The data measured during the run-up and run-down were processed by the short time Fourier transformation (STFT) method. This processing method is commonly used in the analysis of resonance phenomena induced by the start-up and run-down of the machine. STFT is currently built into many diagnostic devices that can simultaneously register the revolutions and measured quantity. It is relatively easy to perform the measurement using the mentioned method if the start-up and possibly the machine rundown can be implemented. The excitation source, in this case, is the machine drive itself or its inertial masses. The non-stationary random process is divided into short time sections; in these sections, the Fourier transform is determined, i.e., the speed change in this section is neglected. On the other hand, other methods can be used to obtain this information, e.g., experimental modal analysis. However, we consider it more demanding, and it is less commonly used among diagnosticians. For this activity, these services are often ordered externally. However, it is true that in addition to resonance areas, we can also obtain other information during this type of analysis, and these are often related to a deeper analysis of the given problem. In our work, we wanted to deal with a simpler tool that is more accessible in diagnostic departments in companies.

**Figure 16:**
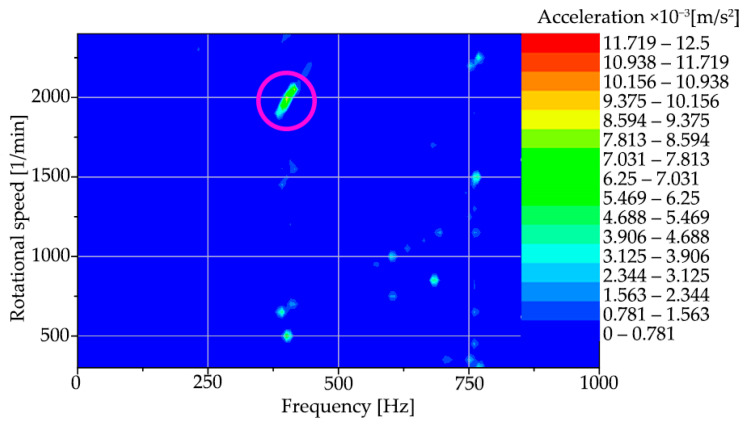
Frequency and speed map for machine start-up with the resonance region marked.

For interpretation, Figure 16 shows a colourmap representing this method of evaluation for region “A”, the machine run-up to steady-state speed.

A resonance region in the vicinity of 400 Hz was identified on the colourmap of the spindle start-up frequencies and speeds, which is built up in the interval of approximately 1950 to 2150 rpm of the spindle.

**Figure 17:**
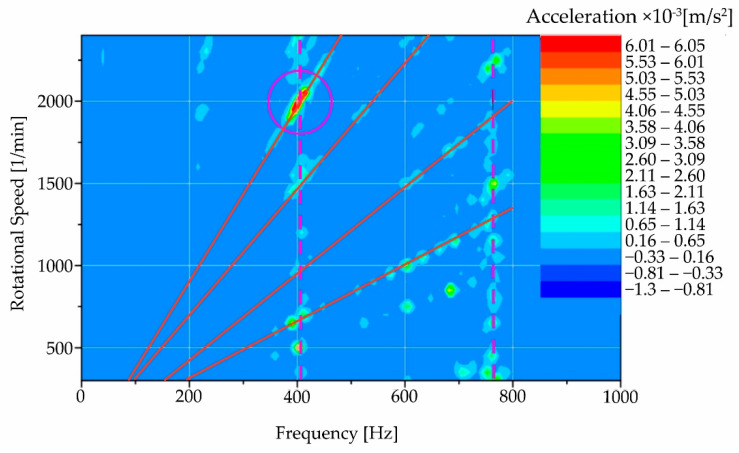
Frequency and speed map for machine start-up with shifted scale.

For a better interpretation of the colourmap (Figure 17), a modified scale of acceleration amplitudes showing the same area as in Figure 16 was used to zoom in. The acceleration amplitudes have a beam-like character; some of them are marked by a red line, corresponding to the increase of the oscillation as a function of the spindle speed. In addition, vertical dashed lines are also marked in the figure. These lines are independent of the speed and represent the resonance action that has been built up by the spindle rotation (or its residual unbalance). The red region in the circle with the maximum oscillation amplitudes is the region that coincides with the region in Figure 16.

**Figure 18:**
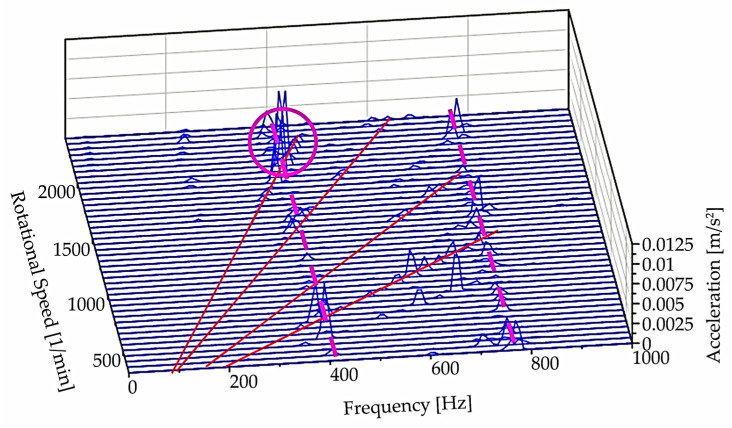
A series of spectra and the beam character of the excitation sources (indicated by red lines).

Color maps represent a three-dimensional representation in the frequency-speed plane, with the axis of amplitudes going toward us. Rotating this three-dimensional representation and removing the color map, we obtain a waterfall-type representation (Figure 18). Where the solid red lines again correspond to frequencies (as in Figure 17) that are dependent on the speed of the grinding wheel, and where the solid lines intersect with the dashed line that marks the position of the natural frequencies, resonance occurs.

The analysis results in detecting a critical region for speeds ranging from approximately 1850 to 2150 rpm. An analysis of the causes of this resonance phenomenon has not been carried out as it requires further measurements. However, with the measurements already made, the user of the equipment has already been informed of the region in which the spindle speed should not be adjusted until the cause of this resonance has been detected.

**Figure 19:**
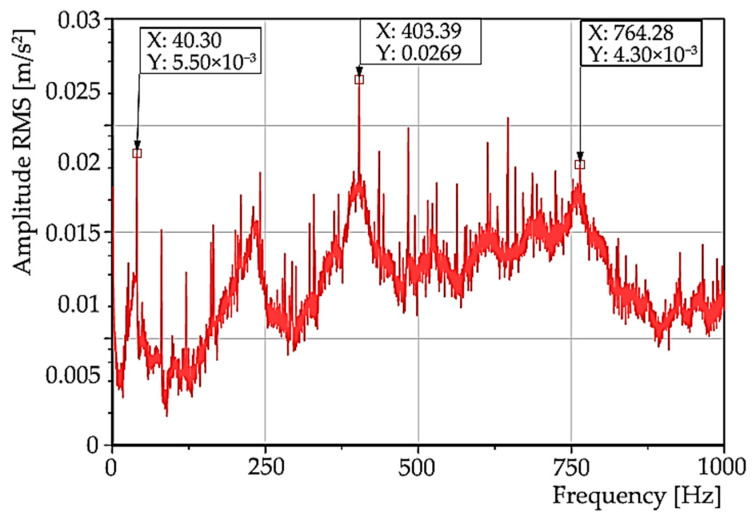
The spectrum of the effective acceleration value in logarithmic scale.

The effective acceleration values for region “B”, i.e., staying at the selected speed, can be plotted on a logarithmic scale (Figure 19). In the spectrum, the frequencies 403 and 764 Hz are marked; these correspond to the resonant frequencies visible on the colourmap. Some multiples of the rotational frequency (periodic occurrence of peaks for n*40.3 Hz, where n is a natural number) are also prominent in the spectrum (Figure 18). The aforementioned appearance of multiples of the rotational frequency in the spectrum is often a symptom of mechanical relaxation in the system. In the following works concerning the analysis of the oscillation of this machine, it will be necessary to detect the cause of this phenomenon.

**Figure 20:**
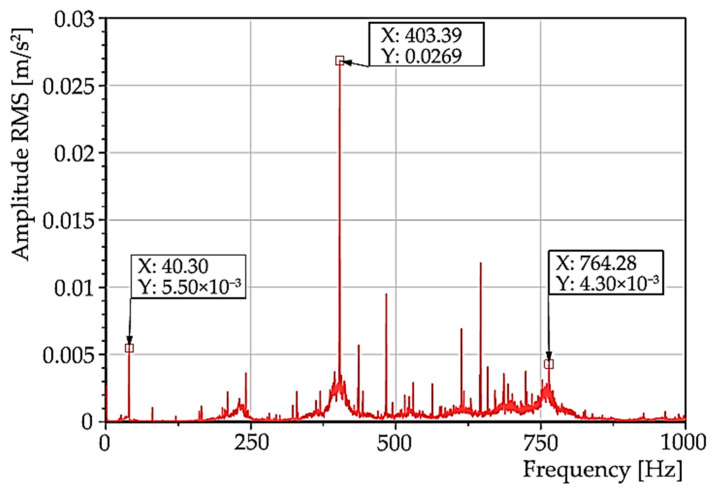
The spectrum of the effective acceleration value on a linear scale with significant amplitude at 400 Hz.

The spectrum of the effective acceleration value on a linear scale (Figure 20) again shows the presence of a significant high amplitude oscillation at 400 Hz, which belongs to the aforementioned resonance region.

For region “C”, the decrease in speed to zero, it was possible to perform the same analysis as for region “A” using the STFT method. However, because the results with processing these areas using the STFT method are identical and sufficiently described for region “A”, only region “A” corresponding to the spindle run-up is presented in the article.

## 4. Conclusions

Identification, analysis, and interpretation of measurement results of the workpiece and grinding wheel oscillations, in particular, due to inappropriately set cutting parameters, are very important steps in vibrodiagnostic research because undesirable oscillations can cause both deterioration of the quality of the machined surface, increased tool wear, increased noise of the machining process, or malfunctions in the operation of the grinder.

The Nomoco grinding machine exhibited a high reject rate in specific grinding regimes, manifested by deviations in geometric dimensions. Since a common cause of this problem is an increased oscillation level, the main objective of the measurement was to find out at which operating revolutions of the support wheel the system will oscillate the least, however, the local extreme of the value course was not determined analytically. In the given experiment, the correct choice of mode was confirmed by checking the geometry that can be achieved at the selected interval of cutting conditions. The required geometry was the roundness of the workpiece. The roundness of the workpiece is achieved empirically in technical practice, as a result of the correct position of the workpiece. In pointless grinding, the workpiece is inserted freely (without clamping and centering) into the gap between the grinding and feed wheel on the support ruler, where the workpiece is carried and ground by the grinding wheel. The workpiece is braked to the required rotation speed by the feed disc. The workpiece is guided between the support ruler and the feed wheel, it is ground during its rotation, while it sits in three straight lines on the grinding wheel and feeding wheel and on the supporting ruler. In addition, an analysis was performed at machine run-up and at steady grinding wheel speed. In the analysis, the region of the speed of this wheel at which resonances occur on the machine was detected. In order to eliminate this resonance happening, a more detailed analysis is required. It was also pointed out that integer multiples of the rotational frequency occur, which is a frequent symptom of mechanical loosening.

Conclusions based on the measurements made and the analysis of the measurement results:

1. The most pronounced oscillations were demonstrated on the ruler through which the workpieces were guided, with the highest values of oscillation amplitudes measured in the vertical direction; these represented an acceleration of 11 m/s^2^.
2. Several measurements of the machine’s operation were carried out, varying the cutting conditions during these measurements. The measurements were intended to give an answer to the question of the sensitivity of the machine oscillation to changing parameters. It was concluded from the processed measurements that the most optimal cutting conditions are at +25 mode, in which the speed of the regulating wheel is increased by 25%.
3. The analysis of the machine run-up waveforms and spectra showed that:
  - There are several speed-dependent excitation sources in the machine; the beamlines of these sources pass through resonant regions that occur in the vicinity of the frequencies 240, 400, and 760 Hz;
  - The most pronounced resonance was in the interval 1950 to 2150 rpm in the frequency range of 400 and 760 Hz;
  - We recommend looking for the excitation source of these events at a frequency of 40 Hz;
  - Higher harmonic components of this frequency appear in the spectra, which is a common symptom of mechanical loosening;
  - If the grinder is to be used in its current state, it is advisable to select mode 25 with the lowest oscillation;
  - If it is necessary to change the speed of the grinding wheel, then it is advisable to avoid the interval 1950 to 2150 rpm where resonance has been detected.

In this article, an original method for evaluating the influence of cutting parameters is presented based on the processing of data from the stationary oscillation region of the workrest during the grinding process, where the recorded data were processed and evaluated by means of statistical characteristics (minimum, lower quartile, median, upper quartile, maximum). The benefit of the evaluation method used is that based on the calculated data, a quick comparison of the different modes can be made. In this case, the inter-quartile range (*x*_0.75_–*x*_0.25_) was used to select the ideal mode.

The experiment also highlights the problems that can occur on the machine due to degradation processes. The run-up and run-down diagnostic method is used to identify these problems. The run-up analysis has shown that there is resonance on the machine. Resonance is also, in the case of the presented results of vibration measurements, evaluated as a technologically significant phenomenon, which can significantly manifest itself at a particular combination of input parameters of the technological process.

We consider the extension of the already used online diagnostic measurements on production machines to be a contribution, whereby the results that characterize the grinding production process are processed using simple statistical procedures.

## Figures and Tables

**Table 1:** Technical data of the CCLD piezoelectric accelerometer.

General Characteristics	Title 2
Frequency range [Hz]	0.3–6000
Temperature [°C]	−54 –… +120
Weight [g]	4.6
Sensitivity [mV/m (with^−2^]	10
Maximum operating level [g]	70
Resonant frequency [kHz]	18
Maximum level (peak) [g]	5000

**Table 2:** Chemical composition of bearing steel for a workpiece.

Steel	Chemical Composition%							
	C	Mn	Si	P	S	Cr	Al	Cu
100CrMnSi6-4	0.93–1.05	1.0–1.2	0.45–0.75	<0.025	<0.03	1.40–1.65	<0.05	<0.03

**Table 3:** Basic cutting conditions for all grinding modes.

Cutting Conditions	Value
Grinding wheel speed [rpm]	1907.2
Support wheel speed [rpm]	20.2
Workpiece speed [rpm]	254
Ruler distance [mm]	13

**Table 4:** Different cutting conditions.

Change of Speed Regulating Wheel [%]	Speed Value after Conversion [rpm]
−25	15.15
−20	16.16
−15	17.17
−10	18.18
−5	19.19
0	20.2
+5	21.21
+10	22.22
+15	23.23
+20	24.24
+25	25.25

**Table 5:** Color coding of the different grinding modes.

Change of Speed Regulating Wheel [%]		−25	−20	−15	−10	−5	0	+5	+10	+15	+20	+25
Color used in boxplot	Grinding 1 *											
	Grinding 2 **											

where * defines the first grinding, ** defines the second grinding.

**Table 6:** Quarterly range percentage value *x*_0.75_–*x*_0.25_.

*x*_0.75_–*x*_0.25_	Axis	−25	−20	−15	−10	−5	0	+5	+10	+15	+20	+25
Operation 1	x	82.94	204.71	114.13	-	87.57	100	74.89	75.37	109.28	107.44	30.18
	y	158.49	154.16	85.12	-	142.87	100	53.50	71.26	86.23	76.91	63.57
	z	125.25	158.49	109.14	-	121.77	100	61.48	66.74	90.61	64.82	59.02
Operation 2	x	85.44	-	110.57	119.31	88.47	100	73.78	71.99	114.12	104.12	30.78
	y	172.90	-	88.67	96.79	151.27	100	56.88	76.35	96.05	79.76	67.1
	z	131.43	-	109	122.52	119.29	100	60.43	64.83	92.92	59.92	59.70

## Data Availability

The data presented in this study are available on request from the corresponding author.
